# SDH-deficient renal cell carcinoma – clinical, pathologic and genetic correlates: a case report

**DOI:** 10.1186/s12894-018-0422-8

**Published:** 2018-11-27

**Authors:** Ravi Kumar, Michael Bonert, Asghar Naqvi, Kevin Zbuk, Anil Kapoor

**Affiliations:** 1 Division of Urology, Department of Surgery, The Ottawa Hospital, University of Ottawa, Ottawa, Canada; 2 Division of Pathology, Department of Pathology and Molecular Medicine, McMaster University, Hamilton, ON Canada; 3 Division of Medical Oncology, Department of Oncology, Juravinski Cancer Centre, McMaster University, Hamilton, ON Canada; 4 Division of Urology, Department of Surgery, Juravinski Cancer Centre, McMaster University, Hamilton, ON Canada

**Keywords:** Succinate dehydrogenase-deficient renal cell carcinoma, Kidney cancer

## Abstract

**Background:**

Succinate dehydrogenase (SDH)- deficient renal cell carcinoma (RCC) is a newly identified rare subtype of RCC, having only gained acceptance from the World Health Organization in 2016. To the best of our knowledge, there are only 55 reported cases worldwide. Here, we report a new case of SDH-deficient RCC.

**Case presentation:**

A 49-year-old male patient was incidentally found to have a large right renal mass. He had no personal or family history of paragangliomas (PGL), pheochromocytomas (PC), or gastrointestinal stromal tumors (GIST). The neoplasm was unilateral and unifocal. He underwent an open partial nephrectomy. Detailed pathological analysis was conducted to confirm the diagnosis. Genetic testing revealed a pathogenic mutation in the *SDHB* gene. He has been followed for 24 months now and has remained well without any evidence of local or distant recurrence. In this report we describe our experience with this diagnosis and review the relevant clinical, pathological, and genetic features.

**Conclusions:**

Without the identification of SDHB deficiency, this patient’s personal and familial predisposition to PC, PGL, GIST and metachronous RCCs may have gone undetected despite his RCC diagnosis. When faced with an eosinophilic RCC, pathologists should routinely search for vacuoles or flocculent cytoplasmic inclusions. When these are present, or in cases of difficult eosinophilic renal tumors, staining for SDHB is recommended. For tumours without adverse pathologic features (i.e. high nuclear grade, coagulative necrosis, or sarcomatoid differentiation) excision alone may be a reasonable option, with the addition of regular surveillance for PC and PGLs in those found to harbor germline SDH mutations.

## Background

Succinate dehydrogenase (SDH)- deficient renal cell carcinoma (RCC) was first identified in 2004 [1]. In 2013, it was integrated into the International Society of Urological Pathology (ISUP) Vancouver classification and in 2016 it was accepted by the WHO organization as a unique subtype of RCC [2].

Succinate dehydrogenase is an enzyme complex consisting of four subunits (SDHA, SDHB, SDHC, and SDHD) that is required for energy metabolism in cells. The majority of patients with SDH-deficient RCC have germline mutations in SDH, with the most commonly mutated gene being *SDHB*, followed by *SDHC, SDHD*, and *SDHA* respectively [3, 4]. Since its first description there have been two cohort studies that have helped identify further clinical and pathological features of this tumour [3, 5]. SDH-deficient RCC is estimated to make up between 0.05 to 0.2% of all renal carcinomas. In patients with a SDHB mutation, the lifetime risk of developing a renal tumour has been estimated at 14% [6]. Patients have developed renal tumours as early as 14 years old, with the mean age estimated around 37 years [3]. SDH-deficient RCC affects both genders, with a slight male predominance. Patients harbouring such mutations are also predisposed to the development of paragangliomas, pheochromocytomas, and gastrointestinal stromal tumors [3, 5]. Given the nascency of SDH-deficient RCC, there are currently no diagnostic or therapeutic guidelines in place to guide management. Here, we report our experience with a new case of SDH-deficient RCC and review the current literature for this rare RCC variant.

## Case

A 49-year-old male presented to the urology clinic after incidental detection of a renal mass. He was asymptomatic, without any hematuria, flank pain, constitutional symptoms, or prior urological history. His past medical history was remarkable for morbid obesity, hypertension, atrial fibrillation, asthma, osteoarthritis, and gastro-esophageal reflux disease. His only prior surgery was a pannulectomy. He reported no relevant family history. Physical examination was unremarkable, except for an obese abdomen and a large ventral hernia. Patient weighed 400 lbs., having previously weighed 500 lbs. His bloodwork showed a hemoglobin of 131 g/L, creatinine of 96 umol/L, and eGFR of 80 ml/min/1.73m^2^.

**Fig. 1:**
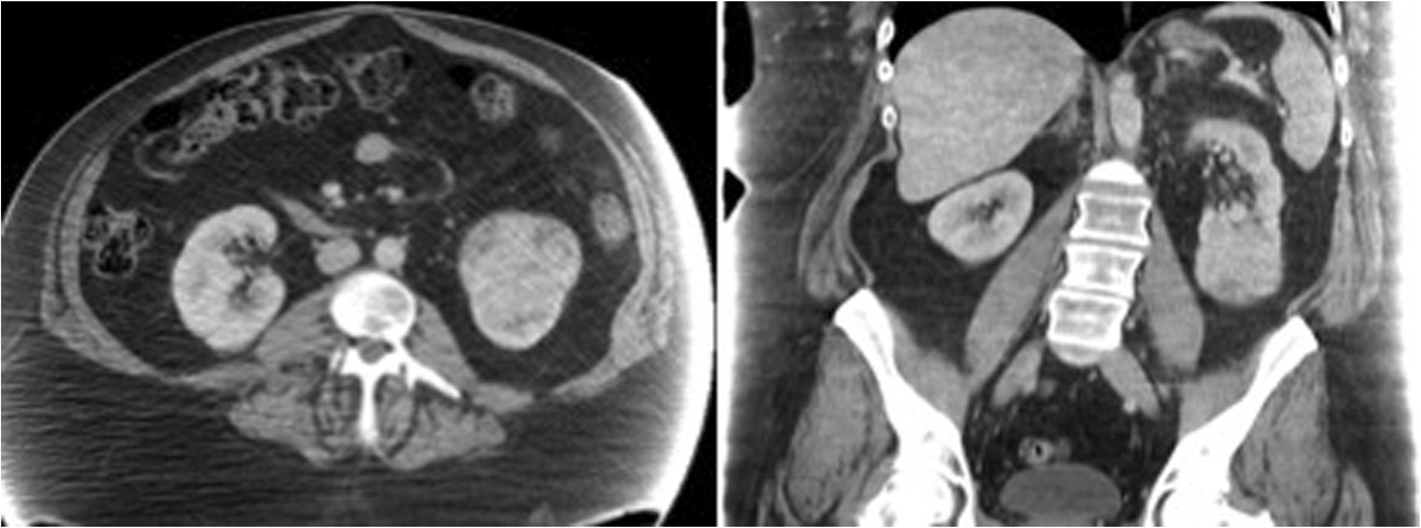
Abdominal computed tomography imaging of the patient shows a large exophytic heterogeneous mass in the lower pole of the left kidney

A CT scan of the abdomen was done as part of a workup for abdominal pain. This revealed a large exophytic heterogeneous mass measuring 9.1 × 9.1 × 10.5 cm in the lower pole of the left kidney (Fig. 1). There was no lymphadenopathy, regional invasion, or distant metastases seen. Bilaterally there were renal cysts without hydronephrosis or hydroureter. A pre-operative CT scan of the chest and bone scan were both negative for metastatic disease. A renogram showed that the large left renal mass was poorly functioning and that there was significant tubular dysfunction affecting both kidneys symmetrically. The function was estimated as 43% on the left and 57% on the right. Review of CT with urology and radiology was suggestive of T2A, N0, M0 renal cell carcinoma. Because of the high likelihood of RCC diagnosis, pre-operative biopsy was offered to the patient, but felt to be unnecessary.

Four months after presentation, he underwent an uncomplicated open left partial nephrectomy. He recovered expectantly post-operatively. The tumor was confined to the kidney with negative surgical margins; pathological stage was pT2a, Nx, Mx.

Since the patient’s surgery, he has been seen in follow up every 6 months with CT imaging. To date, he has remained without evidence of any local or distant tumour recurrence.

### Pathologic correlate

Gross examination revealed a firm-to-rubbery 10 cm tumor located in the lower pole of the left kidney. The tumor was tan brown with areas of hemorrhage and a pale yellow scarred area measuring 3.2 cm.

**Fig. 2:**
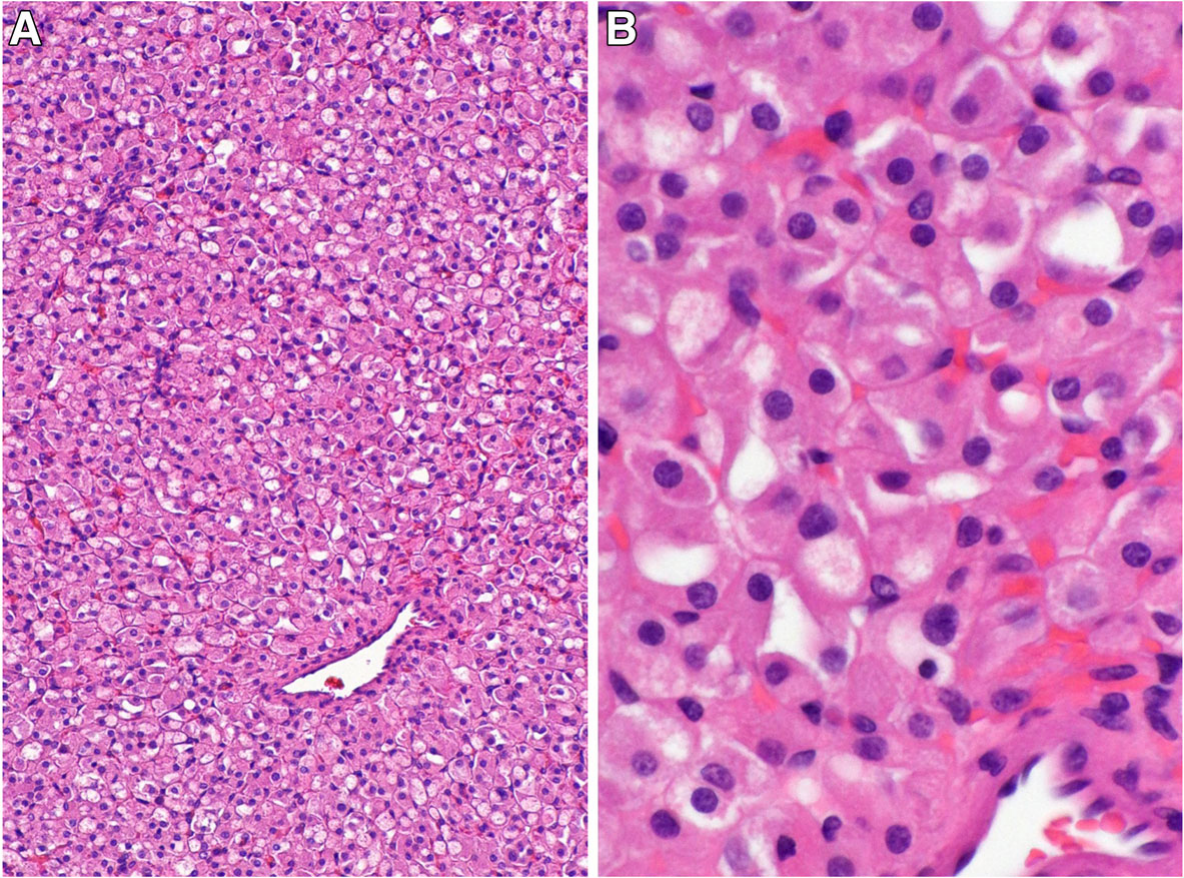
H&E stain. (**a**) 100x original magnification and (**b**) 400x original magnification micrographs showing abundant eosinophilic cytoplasm that is partially vacuolated. The nuclei are round and low grade without no prominent nucleoli or perinuclear halos

**Fig. 3:**
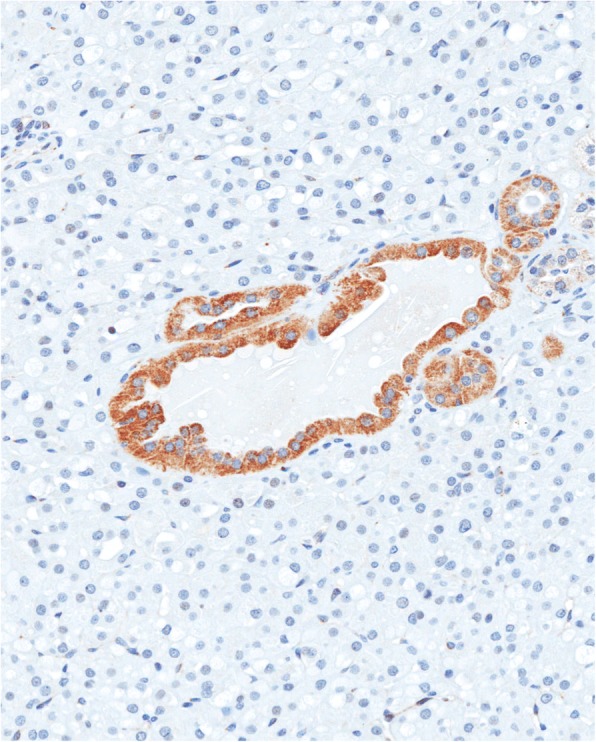
Micrograph showing a section of tumor stained with an SDHB immunostain. The tumour characteristically has lost staining; however, staining is preserved in an entrapped benign tubular structure (200x original magnification)

Microscopic examination showed a solid renal tumor. The cells were intermediate to large in size with partially vacuolated eosinophilic cytoplasms. The nuclei were round (non-resinoid) and without prominent nucleoli or apparent perinuclear halos. The tumor was classified as ISUP nucleolar grade 1 of 4. (Fig. 2). There was no necrosis, sarcomatoid change or increased number of mitotic figures. The tumor cells stained positive for PAX8, AE1/AE3, CAM 5.2, p504S, and EMA. The tumor cells were negative for SDHB, CD117, CK7, CK20, CD10, vimentin, RCC, S100, HMB-45, Melan-A, myogenin, SMA, calretinin, inhibin, DOG1, E-cadherin, and CD56 (Fig. 3).

### Genetic testing and counselling

The absence of SDHB staining by immunohistochemistry confirmed SDH -deficient RCC. Most individuals with SDH -deficient RCC have underlying germline mutations in one of the SDH genes. The patient subsequently underwent genetic counselling and germline mutation analysis of the SDH genes was carried out. This revealed a pathogenic mutation in the *SDHB* gene.

Since there is an increased risk of paragangliomas and pheochromocytomas in SDHB mutation carriers, surveillance for these neoplasms was carried out. A baseline CT scan of the neck/chest/abdomen/pelvis, utilized as the patient’s body habitus precluded MRI scanning, revealed no significant abnormalities aside from post-operative changes post partial nephrectomy. Similarly, baseline 24-h urinary collection for metanephrines and catecholamines was within normal limits. He will continue to undergo annual or biennial biochemical and radiographic surveillance for PC and PGL. Additionally, genetic testing has been offered to family members, who are at risk of inheriting the *SDHB* mutation.

## Discussion and conclusions

Succinate dehydrogenase is required for energy metabolism in cells. It is a part of the Krebs tricarboxylic acid cycle and the mitochondrial electron transport chain. It is composed of four subunits: SDHA, SDHB, SDHC, and SDHD. Anchored by SDHC and SDHD, the catalytic subunits SDHA and SDHB convert succinate to fumarate and pass it on to the next enzyme in the cycle, fumarate hydroxylase (FH) [7]. Mutations in FH are known to underlie the development of Hereditary Leiomyomatosis and Renal Cell Carcinomas (HLRCCs), a hereditary syndrome of RCC [8]. Mutations in SDH had been implicated in familial and sporadic pheochromocytomas and paragangliomas, but not in the development of RCC until recently.

Given the common mitochondrial location of SDH and FH, the possibility of mutations in SDH underlying the pathogenesis of RCCs was explored by Vanharanta et al. in 2004 [1]. By examining a database of patients with symptomatic paragangliomas, they identified 2 members form the same family who had mutations in SDH as well as RCCs (24 and 26 years old respectively). Similarly, in a database of early onset RCC patients they identified a 22-year-old patient with an SDH mutation, who had a RCC and whose mother had a cardiac paraganglioma. These findings suggested a connection between SDH mutations, pheochromocytomas/paragangliomas, and RCCs [1].

Since its first description there have been additional larger studies exploring characteristics of patients with SDH deficient tumours. One of the larger cohorts was the Gill series which included 27 patients with SDH-deficient RCC [3]. From these studies clinical and pathological features were able to be identified.

The mean age of patients was about 37 years, with a slight male predominance of 1.7:1. Approximately 15% of patients had a personal history of gastrointestinal stromal tumours (GISTs), 15% a personal history of paragangliomas (PGLs), 22% had a family history for RCC, 26% had a family history positive for PGLs, and 4% had a positive family history of GISTs [3]. Our patient was a 49-year-old male who did not have a personal or family history of RCC, GISTs, pheochromocytomas, or PGLs.

Pathologically, the colour of SDH-deficient RCC tumours range from tan to red. The majority are well-circumscribed solid lesions with cystic changes being common. The average size of the tumor is about 55 mm. Microscopically, SDH-deficient RCC have solid architecture, eosinophilic cells with clear (flocculent) cytoplasmic inclusions, round nuclei with mildly granular chromatin pattern, and solid architecture. The most characteristic feature is the vacuoles or flocculent cytoplasmic inclusions; however, it may not be prominent in all areas of the tumour. Typically they are low ISUP nucleolar grade but may be sarcomatoid [3]. Our patient’s morphologic findings were largely in keeping the typical findings described for SDH-deficient RCC.

Immunohistochemically, SDH-deficient RCCs are generally positive for PAX8 and EMA, and negative for CK7, CK20, AE1/AE3, and CD117. Immunohistochemical loss of SDHB is a diagnostic requirement. In SDHB-, SDHC- and SDHD-deficient RCCs, tumour cells are negative for SDHB but positive for SDHA. In contrast tumor cells are negative for both SDHA and SDHB in SDHA-deficient RCC [4]. Neuroendocrine markers and epithelial markers are also generally negative. Gill et al. reported that all patients with SDH-deficient RCC who underwent germline mutation testing were found to harbour a pathogenic mutation in one of the SDH subunits. Our patient’s tumour was negative for SDHB and matched the expected immuno-profile. Subsequent germline mutation analysis confirmed a mutation in *SDHB,* the most commonly mutated gene in SDH deficient RCC.

The main differential diagnosis to consider are other renal tumours with eosinophilic cytoplasms such as renal oncocytoma and chromophobe RCC [9]. A full list of differential diagnoses to consider and defining features for each are summarized in Table 1.

**Table 1:** Differential diagnosis of eosinophilic renal cell carcinoma and associated characteristic features

	Macroscopic features	Microscopic features	Immunohistochemistry
Renal Oncocytoma	Classically mahogany brown, well-circumscribed lesion with a central scar	Small solid nests of cells within myxoid or hyalinized stroma. Densely eosinophilic cytoplasm. Nuclei are uniform and round. Prominent nucleoli, typically lacking binucleanation.	Cytokeratin 7: isolated scattered cell staining.
Chromophobe RCC	Usually solitary well-circumscribed grey-beige colored lesion	Solid growth pattern with thin fibrovascular septa. Abundant cytoplasm with prominent cell borders. Nuclei with preserved chromatin and irregular, winkled nuclear membrane.	Cytokeratin 7; usually diffuse staining
Clear cell RCC, eosinophilic variant	Generally golden/yellow color with extensive hemorrhage and necrosis.	Clear cells, although the cytoplasm may be eosinophilic in higher grade tumours. Nested growth pattern. Rich sinusoidal vasculature, often called “chicken wire-like” vasculature.	Positive for CD10, CA-9, EMA, vimentin, and RCC antigen. Negative for CK7 and high-molecular weight keratin. SDHB signal may be weak due to the abundant clear cytoplasm.
TF3 translocation RCC	Yellow-tan with areas of hemorrhage and necrosis	Papillary architecture lined by clear and eosinophilic cells with abundant psammoma bodies. Clear to pale pink fluffy cytoplasm.	Positive for TFE3
SDH-deficient RCC	Tan to red well-circumscribed solid lesions with cystic changes common.	Eosinophilic cells with clear (flocculent) cytoplasmic inclusions, round nuclei with mildly granular chromatin pattern, and solid architecture.	Loss of SDHB is a diagnostic requirement Positive for PAX8 and EMA Negative for CK7, CK20, AE1/AE3, and CD117
Other:	Hybrid oncocytic/chromophobe tumour, tubulocystic carcinoma, papillary RCC, Follicular thyroid-like carcinoma, hereditary leiomyomatosis-associated RCC, acquired cystic kidney disease-associated RCC, epitheloid angiomyolipoma, unclassified RCC, Rhabdoid RCC, MiTF translocation carcinomas		

In the Gill series, the follow up ranged from 0 to 368 months with a mean of 55 months [2]. During that time 9 out of the 27 patients (33%) developed metastatic disease. Two of them occurred after prolonged follow-up (5.5 and 30 years). Four died of metastatic disease at a mean of 18 months after presentation, all of whom had ISUP nuclear grade of 3 or 4 and 3 of whom had coagulative necrosis. Based on this, targeted therapy against vascular endothelial growth factor, mammalian target of rapamycin, and tyrosine kinase have been considered for patients with adverse prognostic factors such as high nuclear grade, coagulative necrosis, or sarcomatoid differentiation. Patients with low-grade tumors showing typical histologic features and an ISUP nuclear grade 2 were usually cured by excision alone. For our patient, a nephron-sparing surgery in the form of left partial nephrectomy was chosen. He had negative margins and no adverse prognostic indicators on pathology. Given these findings no adjuvant treatments were recommended.

Our case illustrates the importance of being familiar with SDH-deficient RCC. The patient described had no personal or family history of RCC, PGL/PC or GIST. The neoplasm was unilateral and unifocal. Finally, the age of onset was not particularly early. Without the identification of SDHB deficiency, this patient’s predisposition to PC/PGL and metachronous RCCs may have gone undetected despite his RCC diagnosis. The identification of an SDH mutation in such cases additionally allows for predictive genetic testing for at risk family members, and subsequent surveillance for RCC and PC/PGL if they harbor the familial mutation. Therefore, when faced with an eosinophilic RCC, pathologists should routinely look for vacuoles or flocculent cytoplasmic inclusions. SDHB immunostaining is useful in eosinophilic renal tumours, especially if the tumor cells are negative for CD117 or there is vacuoles or flocculent cytoplasmic inclusions.

In summary, SDH-deficient renal cell carcinoma is the newest sub-type of RCC that shows distinctive clinical and pathologic features. The tumor can be recognized primarily on the basis of morphology alone, and confirmed with immunohistochemistry. For tumours without adverse pathologic features, excision alone may be a reasonable option, with the addition of regular surveillance for PC and PGLs in those found to harbor germline SDH mutations.

## Abbreviations

FH: Fumarate hydroxylase
GIST: Gastrointestinal stromal tumors
HLRCCs: Hereditary Leiomyomatosis and Renal Cell Carcinomas
HOCT: Hybrid oncocytic/chromophobe tumour
ISUP: International Society of Urological Pathology
PC: Pheochromocytomas
PGL: Paragangliomas
RCC: Renal cell carcinoma
SDH: Succinate dehydrogenase
WHO: World Health Organization
